# The First Wedding

**Published:** 1880-07

**Authors:** 


**The First Wedding.**—We like the snort courtships, and in this Adam acted like a sensible man—he fell asleep a bachelor, and awoke to find himself a married man. He appears to have popped the question almost immediately after meeting Miss Eve, and she, without flirtation or shyness, gave him a kiss and herself. Of that first kiss in the world we have had our own thoughts, however, and sometimes, in a poetical mood, wished we were the man that did it. But the deed is done—the chance was Adam's, and he improved it. We like the notion of getting married in a garden. Adam's was private. No envious aunts and grunting grandmothers. The birds of the heavens were the minstrels, and the glad sky flung its light on the scene. One thing about the first wedding brings queer things to us in spite of its scriptural truth. Adam and his wife were rather young to marry; some two or three days old, according to the sagest elder; without experience, without a house, a pot or kettle; nothing but love and Eden.

**Marriage.**—Marriage is to a woman at once the happiest and saddest event of her life; it is the promise of future bliss, raised on the death of present enjoyment. She quits her home, her parents, her companions, her amusements—every thing on which she has hitherto depended for comfort, for affection, for kindness, and for pleasure.

The parents by whose advice she has been guided—the sister to whom she has dared to impart the very embryo thought and feeling—the brother who has played with her, by turns the counselor and the counseled, and the younger children to whom she has hitherto been the mother and playmate—all are to be forsaken at one fell stroke—every former tie is loosened—the spring of every action is changed; and she flies with joy in the untrodden paths before her, buoyed up by the confidence of requited love, she bids a fond and grateful adieu to the life that is past, and turns with excited hopes and joyous anticipation to the happiness to come. *Then woe to the man who can blight such fair hopes*—who can treacherously lure such a heart from its peaceful enjoyments, and watchful protection of home—who can, coward-like, break the illusions which have won her, and destroy the confidence which love had inspired.

**Woe to him who has too early withdrawn the tender plant from the props and stays of moral discipline, in which she has been nurtured, and yet makes no effort to supply their places; for on him is the responsibility of her errors—on him who first taught her, by his example, to grow careless of her duty, and then exposed her, with a weakened spirit and unsatisfied heart, to the wild storms and the wily temptations of a sinful world.—** *Anon.*

